# Prevalence of *Schistosoma mansoni* infection and the therapeutic efficacy of praziquantel among school children in Manna District, Jimma Zone, southwest Ethiopia

**DOI:** 10.1186/s13071-016-1833-6

**Published:** 2016-10-24

**Authors:** Mitiku Bajiro, Daniel Dana, Mio Ayana, Daniel Emana, Zeleke Mekonnen, Belay Zawdie, Asfaw Garbi, Ashenafi Kure, Ahmed Zeynudin

**Affiliations:** 1 Department of Medical Laboratory Sciences and Pathology, Jimma University, Jimma, Ethiopia; 2 Departement of Biomedical Sciences, Jimma University, Jimma, Ethiopia; 3 Public Health Laboratory, South Nations Nationalities and People’s Regional State Health Bureau, Hawassa, Ethiopia

**Keywords:** *Schistosom mansoni*, Kato-Katz, Praziquantel, Prevalence, Intensity, Cure rate, Egg reduction rate

## Abstract

**Background:**

Intestinal schistosomiasis is one of the neglected tropical parasitic diseases caused by *Schistosoma mansoni.* Currently, the control measures for the disease are mainly based on mass drug administration (MDA) with praziquantel (PZQ) targeting the school-age children. In Ethiopia, the potential foci for schistosomiasis and therapeutic efficacy of PZQ among school-age children remain poorly explored. Therefore, we determined both the prevalence and intensity of *S. mansoni* infection and the therapeutic efficacy of PZQ among school children in the Manna District (new foci for *S. mansoni*), Jimma Zone, southwest Ethiopia.

**Methods:**

A cross-sectional study was conducted among the school children aged between 6 and 18 years in three primary schools in Manna district from March to April 2014. For diagnosis of *S. mansoni*, a single stool sample was obtained from each child and processed using single Kato Katz and examined under light microscopy. A questionnaire was used to collect demographic information of the school children participated in the study. School children excreting eggs of *S. mansoni* were administered with 40 mg/kg of PZQ and re-examined after three weeks post-treatment. The therapeutic efficacy of PZQ against *S. mansoni* was evaluated by means of cure rate and egg reduction rate.

**Results:**

The overall prevalence of *S. mansoni* among the school children in the three primary schools in Manna District was 24.0 %. Higher prevalence was recorded for males 25.6 % (61/238) than for females 22.5 % (59/262). Majority (27.5 %) of infection intensity was light with mean faecal egg count (FEC) of 202 eggs per gram (EPG). The therapeutic efficacy of PZQ at a dose of 40 mg/kg was highly efficient (cure rate of 99.1 % and egg reduction rate of 99.9 %) among the school children in the three primary schools in Manna District.

**Conclusions:**

The school children in the three primary schools of Manna District, Jimma Zone were at moderate risk of the morbidity caused by *S. mansoni* (prevalence > 10 % and < 50 % according to WHO threshold), and hence a biannual MDA with PZQ is required. PZQ available on the local market was found efficient and can be recommended for individual treatment in absence of MDA. The therapeutic efficacy of PZQ at 40 mg/kg against *S. mansoni* was high in the study area.

**Electronic supplementary material:**

The online version of this article (doi:10.1186/s13071-016-1833-6) contains supplementary material, which is available to authorized users.

## Background

Intestinal schistosomiasis is a chronic parasitic disease caused by the trematode *Schistosoma mansoni* [1]. It is one of the neglected tropical parasitic diseases (NTDs) that causes severe morbidity and mortality among susceptible segments of the population. Its importance in terms of socioeconomic and public health in tropical and subtropical countries is high. In tropical developing countries about 779 million people were estimated to be at risk of infection (85 % in Africa), 207 million people are infected (> 97 % in Africa) and 120 million people diseased (20 millions severely) [2, 3]. The annual mortality is estimated to be 15,000–280,000 and 1.7–4.5 million disability adjusted life years are lost due to disease [4].

In Africa, 31 countries including Ethiopia share the great burden of schistosomiasis and millions of people have been suffering from the disease. The burden of schistosomiasis due to *S. mansoni* is 28.8 million in Nigeria, 19 million Tanzania, 15.2 million Ghana, 14.9 million Congo and 13.2 million Mozambique [3, 5]. In Ethiopia, the endemicity of intestinal schistosomiasis has long been established and estimates made in the early 1980s documented the number of people at risk of infection as 18 millions [6]. Several epidemiological studies indicate that intestinal schistosomiasis due to *S. mansoni* infection is widely distributed in different parts of Ethiopia with prevalences as high as 90 % in school-age children [7].

Children in the developing countries live in areas with poor sanitation and most often spend time swimming or bathing in the water bodies contaminated with cercariae, the infective stages of schistosomes [8]. The hygiene and playing behavior in water bodies increases the risk of being infected by *S. mansoni* [9].

In the past, the preventive measures of schistosomiaiss largely focused on decreasing or interrupting transmission of the infection; however such measures have not been continued due to high operation cost/difficult implementation logistics [5]. The advent of inexpensive, efficacious and safe drugs has made chemotherapy the most cost-effective strategy thereby shifting control emphasis to disease control [10].

Oxamniquine, metrifonate and PZQ are the main drugs that have been used widely for control of schistosomiasis [11, 12]. Among these anti-schistosomial drugs, PZQ is a broad spectrum drug that is effective against all species of *Schistosoma*. It is administered orally at a standard single dose of 40 mg/kg body weight and well tolerated. The side effects of the drug are mild and transient, and mostly related to the gastrointestinal tract effects, such as abdominal pain, nausea, vomiting, anorexia and diarrhea [13].

The efficacy of the drug is considered as sufficient when the cure rate (CR) is 60–90 % and egg reduction rate (ERR) is > 90 %, while facilitating patients’ compliance especially among children [14, 15]. Most of the time population treatment with PZQ produces CR of over 70 %. However, efficacy of treatment is influenced by a number of factors, such as the epidemiological situations and expands on the prevailing ecological conditions which may affect PZQ efficacy [16]. Efficacy of PZQ decreases with pre-treatment intensity of infection, number of pre-patent infections, diagnostic sensitivity and age of the treated individuals [17]. Moreover, poor drug quality and poor patient compliance may also negatively impact the effectiveness of the treatment, and even optimal timing at which treatment is evaluated also affects the outcome of the treatment [18].

Some studies conducted on the efficacy of PZQ against *S. mansoni* tested in Senegal reported CR as low as 18 % raising the fear about the effectiveness of the drug [17]. However, there have been reports of *S. mansoni* resistance to PZQ from studies carried out in Egypt with failure to CR of 1.6 % of those infected with *S. mansoni* in a Nile Delta village [19] and Kenya [20]. There are reports of low CR of 73.6 % and ERR of 68.2 % of PZQ in southern part of Ethiopia [21].

Our study area is not yet included in the epidemiological map of *S. mansoni* in Ethiopia and there are various reports providing evidence for parasite occurrence from the local health offices in the district. To the best of our knowledge, the epidemiology of *S. mansoni* and the therapeutic efficacy of PZQ have not been evaluated in the study area. Therefore, we determined the prevalence and intensity of *S. mansoni* infections and evaluated the therapeutic efficacy of PZQ against *S. mansoni* among the students of Manna District, Jimma Zone, southwest Ethiopia.

## Methods

### Study area

The study was conducted between March and April, 2014 among students of three primary schools namely *Kore konjo*, *Wollo sefar* and *Saye odo* in Mana District, Jimma Zone, Oromia Regional State, southwest, Ethiopia. The district is located 382 km away from the capital city of the country and 32 km away from Jimma Town in Jimma Zone. The district is located at an average altitude of about 1450 m above sea level. It is generally characterized by warm climate with a mean annual maximum temperature of 25 °C and a mean annual minimum temperature of 18 °C. The annual rainfall ranges from 1138 to 1690 mm (Report document 2013/2014 of Jimma Zone administration). There are different water sources in the district which the population frequently used for domestic purposes and thus could be potential risk factor for infection with *S. mansoni*.

### Study population

Study populations were all school children enrolled in three primary schools of Manna district during study period. In each school, we stratified students according to three age groups (age 6–9 years, age 10–14 years and age 15–18 years). Five hundred students were screened to include at least 100 *S. mansoni*-infected children at baseline screen based on WHO guideline and other literature for assessing the efficacy of anti-helminthic drugs against schistosomiasis and soil-transmitted helminthiases [15, 22].

### Sampling, study design and sample processing

Simple random sampling technique was used to select the study participants based on their name list from their grades. School-based cross-sectional study was employed among school children of three primary schools in the Manna District. Stool samples were collected using dry, clean, labelled plastic containers. Kato-Katz thick smear was prepared from each stool sample for the detection and quantification of the eggs of *S. mansoni* and other soil-transmitted helminths (STH). The quantity of *S. mansoni* eggs was determined before and after treatments to determine the intensity of infection. Before the administration of PZQ, all *S. mansoni*-infected students were provided biscuits and a cup of tea. Students who were treated with PZQ were followed by senior health officer and nurse for four hours and those who vomited within two hours after oral administration of the drug were excluded from the analysis.

### Praziquantel efficacy

In post-treatment, a Kato-Katz thick smear was processed for each specimen to determine CR and ERR. Thus, efficacy of 40 mg/kg PZQ against *S. mansoni* was determined exactly on day 21 post-treatment and its efficacy were assessed as ERR (%) as follows [15]:

$$ \mathrm{ERR}\ \left(\%\right)=100 \times \left(1-\frac{\mathrm{Arithmetic}\ \mathrm{mean}\ \mathrm{of}\ \mathrm{egg}\ \mathrm{counts}\ \mathrm{at}\ \mathrm{follow}\hbox{-} \mathrm{up}}{\mathrm{Arithmetic}\ \mathrm{mean}\ \mathrm{of}\ \mathrm{egg}\ \mathrm{counts}\ \mathrm{at}\ \mathrm{base}\ \mathrm{line}}\right) $$

The indicator of choice for drug efficacy is ERR. PZQ efficacy is satisfactory when ERR ≥ 90 % with the reference value ≥ 90 %; doubtful when ERR is lower than the reference value by less than 10 %; and reduced if ERR is inferior by value of at least 10 % points than the reference value [15].

### Data processing and analysis

Data were coded, entered and cleaned by using EPIIFO. Data processing and analysis were carried out using SPSS version 20.0. CR was calculated as the ratio of the number of study participants who were negative after treatment to the number of study participants who were positive before treatment and who completed the study and ERR was calculated based on arithmetic means as described above. Infection intensity of S*. mansoni* was provided as geometric mean.

### Data quality assurance

Refreshment training was given to data collectors and laboratory technicians about Kato-Katz smears by experienced personnel in the field. During data processing, the quality of data was assured by coding and double entry. From both positive and negative Kato-Katz smears, 10 % were randomly selected and re-read by two independent medical laboratory experts who were blinded to the primary result. Moreover, fresh working solution of malachite-green was used routinely to maintain the quality of the smear.

## Results

### Socio-demographic characteristics of study participants

A total of 500 students (238 males and 262 females) were involved from three primary schools selected in Manna District. The largest number of study participants, 379 (75.8 %) was from the age group 10–14 years.

### Prevalence and intensity of *S. mansoni* infection

**Fig. 1:**
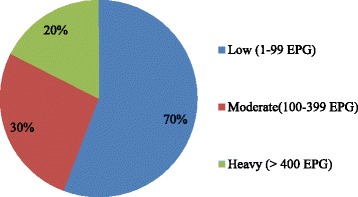
Infection intensity of *S. mansoni* among school children in Manna District, Jimma Zone, southwest Ethiopia, 2014

The overall prevalence of *S. mansoni* among students was 24 % (120/500). The prevalence was 25.6 % (61/238) and 22.5 % (59/262) for the male and female students, respectively. The prevalence ranged from 7.6 to 41.4 % among the schools with the highest prevalence 41.4 % (41/99) in *Kore Konjo* School (Table 1). Majority of the infection intensity was classified as low with maximum EPG of 1848 (Fig. 1).

**Table 1:** Socio-demographic characteristics and prevalence of *S. mansoni* among school children in three primary schools of Manna District, Jimma Zone, southwest Ethiopia, 2014

Variables	*S. mansoni* infection status		Total (%)
	No. of positive (%)	No. of negative (%)	
Sex			
Male	61 (25.6)	177 (74.4)	238 (47.6)
Female	59 (22.5)	203 (77.5)	262 (52.4)
Age (years)			
6–9	8 (10.4)	69 (89.6)	77 (15.4)
10–14	98 (26.1)	278 (73.9)	376 (75.2)
15–18	14 (29.8)	33 (70.2)	47 (9.4)
Schools			
Kore Konjo	41 (41.4)	58 (58.6)	99 (19.8)
Saye Odo	66 (28.6)	165 (71.4)	231 (46.2)
Wollo Sefar	13 (7.6)	157 (92.4)	170 (34)

### The therapeutic efficacy of praziquantel

Among the study participants, 120 students were infected with *S. mansoni* and treated with 40 mg/kg PZQ at baseline screening. Of the 120 infected students, five were excluded from the analysis of PZQ efficacy after treatment with 40 mg/kg PZQ. Two of them vomited immediately after 20 min of drug administration and three refused to provide stool on the day of post-examination. The CR of PZQ against *S. mansoni* infected school children was 99.1 %. The ERR of 40 mg/kg PZQ was found to be 99.9 %. ERR and CR had no statistically significant association with age of the school children and intensity *S. mansoni* infection (*χ* ^2^ = 1.74, *P* = 0.424) (Table 2).

**Table 2:** Cure rate of PZQ against *S. mansoni*-infected school children in Manna District, Jimma Zone, southwest Ethiopia, 2014

Age group	Cure rate [*n* (%)]		Total [*n* (%)]	*χ* ^2^	*P*-value
	Cured [*n* (%)]	Uncured [*n* (%)]			
6–9 years	6 (85.7)	1 (14.3)	7 (6.1)	1.71	0.424
10–14 years	94 (100)	0	94 (81.7)		
15–19 years	14 (100)	0	14 (12.2)		
Total	114 (99.1)	1 (0.9)	115 (100)		

## Discussion

In the present study, the overall infection with *S. mansoni* among students of three primary schools in Manna District was 24 %. The therapeutic efficacy of PZQ at 40 mg/kg body weight against *S. mansoni* infection in the study area had 99.9 % ERR and 99.1 % CR. Majorities of infection intensities and the mean intensity of infection were low.

The prevalence rate in the present study was higher than those reported from Brazil (14.4 %), Nigeria (4.6 % and 12.6 %) and Ghana (19.8 %) [23–26]. Similarly, our findings were higher than two previous investigations conducted in Ethiopia including Tigray (5.95 %) and Jimma (2.1 %) [27, 28]. The difference could be due to the study period and the method of laboratory diagnosis employed.

However, there are reports with higher prevalence from northwestern Tanzania (64.3 %) [29], and from southern Tigray (73.9 %), Gonder (89.9 %), Wondo Genet (74.9 %) and Wollega (67.6 %) from Ethiopia [7, 21, 30–32]. The difference might be due to long time endemicity of the parasite in these study areas than the current emerging foci of our study area, the study design employed and ecological differences.

There is almost comparable report from Uganda (27.8 %) [33] and other similar studies from Ethiopia including Mekelle (23.9 %) [34] and southeast of Lake Langano (21.2 %) [35].

The infection intensity in our study indicated low infection levels comparable with findings from Timuga and Waja from Tigray [36] and Mekelle City [34]. However, moderate infection intensities were reported from Wondo Genet [21] and Wollega [32] in Ethiopia. The difference may be explained by the frequency of students’ contact with contaminated water-bodies and the burden of the adult worms hosted.

In the current study area, PZQ at 40 mg/kg had higher efficacy when compared with the reports from Senegal (CR = 42.5 % and ERR = 70.7 %) [37] and Nigeria (CR = 49.4 % and ERR = 57.1 %) [14]. This difference could be due to baseline infection intensity, duration of post-treatment, presence of immature stages of the parasite. Moreover, the new emergence of *S. mansoni* in the study area and the lack of previous exposure of school children to PZQ treatment as mass drug administration may contribute to the high efficacy. Previous efficacy study carried out at four weeks of post-treatment in northeast Ethiopia have reported lower CR (83.2 %) [38]. Moreover, lower CR (73.6 %) and ERR (68.2 %) were reported from other studies undertaken in Wondo Genet, Ethiopia [21]. The difference might be due to the intensity of infection, brand of PZQ used, geographical variation, and the presence of immature stages of the parasite. On the contrary, the efficacy of PZQ at 40 mg/kg in the present study was higher compared to the study reported from Senegal (CR 18–38 %) [39], Niger (ERR 55.2–60.2 %) [40] eventhough the duration of post-treatment was similar to that of our study (three weeks). The variation of PZQ efficacy might be due to the infection intensity and geographical location.

The efficacy of PZQ at 40 mg/kg (CR = 99.1 % and ERR = 99.9 %) in the present study is almost comparable to the reports from South Africa (CR = 100 %) [41], Sudan (CR 89–92.1 % and ERR 96.4–99.4 %) [42], Egypt (CR 73.3–92.8 %) [19] and Kenya (ERR = 92.6 % [43]. Moreover, there are two reports from Ethiopia including Tigray, Timuga and Waja (CR of 93.44 and 88.9 %) [36], Wollega (CR of 80.9 % and ERR of 99.5 %) [32], respectively, from Ethiopia at different post-treatment durations with comparable efficacy. Furthermore, the efficacy of PZQ at 40 mg/kg among students in present study is comparable with the findings from Senegal (CR = 93 % and ERR = 90 %) [18], Cameroon (CR = 95.3 %) [22], and Ethiopia (CR = 94 % and ERR = 97 % [44] with the same post-treatment duration.

## Conclusions

The students in the three primary schools of Manna District, Jimma Zone were at moderate risk of the morbidity caused by *S. mansoni* (prevalence > 10 % and < 50 % according to WHO threshold), and hence a biannual MDA with PZQ is required. PZQ available on the local market revealed efficiency and can be recommended for individual treatment in absence of MDA. The infection intensity of *S. mansoni* among the school children in the current study was light. Among school children, the age group 10–14 years is the most affected. PZQ at 40 mg/kg has 99.9 % ERR and 99.1 % CR. Therefore, the therapeutic efficacy of PZQ at 40 mg/kg against *S. mansoni* was high in the study area.

## Additional file

Additional file 1: Consent form. (DOCX 16 kb)

## Abbreviations

CR: Cure rate
ERR: Egg reduction rate
MDA: Mass drug administration
NTD: Neglected tropical diseases
PZQ: Praziquantel
STH: Soil-transmitted helminth
WHO: World Health Organization
