# Supplementary material for: Prevalence of Schistosoma mansoni infection and the therapeutic efficacy of praziquantel among school children in Manna District, Jimma Zone, southwest Ethiopia
Source: Parasit Vectors. 2016 Oct 24;9:560. doi: 10.1186/s13071-016-1833-6 (PMC5075754; doi:10.1186/s13071-016-1833-6)
Supplement: Additional file 1: — Consent form. (DOCX 16 kb) [file 13071_2016_1833_MOESM1_ESM.docx]

**Additional file 1.**

**Consent form**

**Explanation on procedures and conditions of agreement**

We are from Jimma University, college of public health and medical science. We are here to study the problem of the disease called Shistosomiasis. The objective of the study is to determine efficacy of PZQ against *S. mansoni* in school children and subsequently to help in the initiation of the control programs. We are asking you and the guardians of the children to allow the students to participate in this study. Examination will involve only laboratory examinations stool samples. We will give the students stool cup to collect their samples. Then we take the stool samples to Jimma University Medical Laboratory Science and Pathology Laboratory to examine the stool by Kato-Katz parasitological examination methods to detect the presence or absence of intestinal parasites giving emphasis to *S. mansoni* and students who are positive for *S. mansoni* will be treated with PZQ 40mg/kg and students who are positive for other parasite will be treated by albendazole 400 mg. However, if in case any problems arise during treatments with PZQ we make necessary care. And those students who take PZQ we will again collect stool sample three weeks after treatments to determine the efficacy of the drug against *S. mansoni*. If you have understood the explanation well enough, we are asking you to allow your child to participate in this study. If you decide to volunteer, your child involve we are kindly ask you to put your signature as illustrated below.

I, the undersigned, will like to confirm that, I give consent to my child participate in this study; it is with a clear understanding and recognition of:

A. The objectives of the intended study,

B. The procedures of diagnosis and treatment

I confirm that the proposal has been explained to me in the language I am fluent and conversant.

Name of the guardians: ________ Signature: ____________ Date: ________________

Name of researcher: ___________ Signature: ___________ Date: _________________

**Guca wadda (Translated to local language (Oromifaa)**

Gucawaligalte matiin baraata qorraannoo kessaatti hiramchisiisuuf fedhii qabachuu isaan ittin walgalte isaan ibsan.

Nutti kan dhufne Jimma Uniiverisitti Koollejjii Fayyaa Hawaasa fi Saayinsii Meedikaala irraatti.Nutti kan asii dhufneef rakkina wa’ee dhukkuba bilaarziyaa fi dawwaa (piraaziku’antilii) isaa hangam akka maxxantuu dhukkuba kan fiduu dhabamsiisuu qorraachuudhaaf. Kaayyoon qorraanoo kana babal’ina fi dawwaan dhukkubaa kanaaf kennamuu hamaam akka inni rammoo dhukkuba kana fiduuf dadaarbinsaa isaaf sabaaba guddaa fi ijoo kan ta’e buupha isaa balleessuu ykn immoo hir’isuu qorraachuudhaaf. Rammoon kunniif kanneen biraa yoo baraataan qaba ta’ee akka baraatan sirritti kutaa keessa ta’ee barumsaa isaa sirritti hin hordoofneef barumsaa sirritti hin hubaneef sabaaba ta’an keessaa isaa tokkoodhaa. Kanaaf nutii waan barbaadnu qorraanoo labooratorii geegisinee baraata dhibee kana qabuuf akka inni yalamuuf maxxantuu irra bilisaa gochuudha. Nutti kan isini gaffaanu maatin barraatotaa ijoollen keessaan akka isaan qorraannoo kana keessati akka hirmataan isinii iyyaama akka nuuf keenitan isinii gaffaana. Qorraannoo kana keessatti ijoollee keessaan hiramachisuu ykn hirmachisuu dhisuu ni dandeesu. Qorraannoo kanaf kan nuti barbaanuu ergaa isiin hirmachisumma baraata(mucaa) keessan beeksiistanii booda boolii furdaa akka baraatan keennuu gaffanaa isaa booda boolii kana funnee gara Jimmaa Uniiveesittiti funne ergaa fidnee booda qorraannoo boolii geggeessudhaan akkaa maxxaantuu dhukkubaa bilaarziyaa fiduu fi kanneen rammoo dhukkubaa garaa keessaa fidaan biraan jiraachuu fi jiraachuu dhibuu isaan qorraannaa. Boolii funne gara Jimmaa Uniivesiitti fidnuu kana qorraanoo boolii geggeesuuf qofaaf fayyadamna. Firrii qorraannoon boodaa baraatan buuphaa dhukkubaa bilaarziyaaf sabaaba ta’uu boolii isaa keessaatti argameef buphaan rammoole gara biraan gaara isaan keesaatii argamaaniif dawwaa piraaziku’antilii jedhamuu kanbilaarziyaaa jeessuu fi albeendazoolii kan rammoolee biraa ajeessuu hakimootaa bufaata fayya Qorree keessaa hojetaaniin baraatotni qaban akka yalaaman tasiiffamaa. Adeemsaakana keessaatti waan akka gaara cininuu yoo jiraate baraatan dawwaa kana fudhaate hangaa sa’attii afurii bufaata fayyaa turee ilaalama.

Qorraannoo booliifis ta’ee kan dawwaa baraatan itti yaalamu matiin baraata irraa kaffaltiin eggaamuu tokkoollee hinjiruu kan isinii irraa eggamuu akka baraatan(mucaan) keessaan keessatti hirmaachuu danda’an iyyaamaa keessan mallaattoo keessaaniin mirkaneessuu qofaatuu isinii irraa eggamaa.Firriin (gabaasini) qorraanoo kan odeeffanoo wa’ee enyumaa baraata kan ibsuu hinqabu. Fakkeenyaaf maqaa baraata, nannoo baraata kan ibsuuf kkf akka inni hin qabnee ofitti amanmuumadhaan isin beeksisuu barbaadna. Yoo barbaachisumaa isaa sirriiti hubaatan itti ammantanii ta’ee akka baraatan(mucaan) keessaan qorraanna kan keesaatti akka hirmatuuf isin ni gafaana. Hirmachuuf hirnachuu dhisuu baraata/ttu(mucaa) kessaan akka mallattoo keessaniin nuu mirkaneesitaan isin ni gafana .

Anni kan armaan gaditti mallateesse, fedhii kootiin baraata/ttuu(mucaa) koo qorraanoo kana kessaatti akka hirmaatu/ttuuf gutumma gutuutti kaayyoon qorranichaa, adeemsaa qorraanoof fayyidaa inni qabu hubadhee iyyaamuu koo mallaattoon kottiin beeksiisaa.

Maqaa abbaa/ hadhaa baraataa ____________________________

Mallattoo___________________________________________

Guyyaa________________________________________

Maqaa abba qorraannoo geggeessu_______________________________

Mallattoo___________________

Guyyaa____________________________
